# Hypo-Hydroxymethylation of Nobox is Associated with Ovarian Dysfunction in Rat Offspring Exposed to Prenatal Hypoxia

**DOI:** 10.1007/s43032-022-00866-6

**Published:** 2022-03-07

**Authors:** Changfang Yao, Likui Lu, Yiting Ji, Yingying Zhang, Weisheng Li, Yajun Shi, Jinliu Liu, Miao Sun, Fei Xia

**Affiliations:** 1 Reproductive Medicine Center of the First Affiliated Hospital of Soochow University, Suzhou, 215006 Jiangsu China; 2 Obstetrics of the Third Affiliated Hospital of Soochow University, Changzhou, 213000 Jiangsu China; 3 Institute for Fetology, The First Affiliated Hospital of Soochow University, Suzhou, Jiangsu China

**Keywords:** Prenatal hypoxia, Ten-eleven translocation, Fetal origins of adult disease, Newborn ovary homeobox gene, Ovarian

## Abstract

Prenatal hypoxia (PH) is a common feature of a suboptimal intrauterine environment affecting the development of fetuses. Whether PH leads to abnormal ovary development is not yet clear. This study investigated ovarian function in offspring exposed to PH and the potential underlying molecular mechanisms. SD female rats (*n* = 12 per group) at 9 weeks of age were housed in individual cages (21% O_2_). After the pregnant rats were exposed to hypoxia (10.5% oxygen) from embryonic day (E) 5 to E21, PH offspring were generated. All animals maintained normoxia during lactation. The number of follicles was counted in female offspring at 3 months under an optical microscope. The expression of Nobox, Gdf9, and Tets was detected by quantitative real-time polymerase chain reaction (PCR) and Western blot. Global DNA hydroxymethylation was measured by dot blot. The hydroxymethylation level of the Nobox gene was evaluated with an NGS-based multiple targeted CpG hydroxymethylation analysis method. Body weight and ovary weight were significantly decreased in the PH group compared with the control group. PH offspring have abnormal estrous cycle, decreased serum anti-Mullerian hormone (AMH), and increased serum follicle-stimulating hormone (FSH), and follicular atresia, which are consistent with the clinical manifestations in patients with ovarian dysfunction. In terms of mechanism, the expression of Nobox was significantly decreased in the PH group. Subsequent high-throughput sequencing results showed that the level of hydroxymethylation in the candidate region of the Nobox gene was reduced. Cultured cells treated with hypoxia exhibited lower levels of both 5hmC and Nobox, while vitamin C, a coactivator of Tets, rescued hypo-hydroxymethylation and increased the expression level of Nobox. This study indicated that PH could cause hypo-hydroxymethylation of Nobox through epigenetic regulation and may consequently contribute to ovarian dysfunction in adult rat offspring.

**Supplementary Information:**

The online version contains supplementary material available at 10.1007/s43032-022-00866-6.

## Introduction

The intrauterine environment plays an important role in shaping fetal development and impacting later health [1]. Prenatal hypoxia (PH) is fetal distress which is caused by three factors [1–3], including maternal factors (such as maternal nutrition, maternal smoking, and maternal hypertension), fetal factors (such as umbilical cord around neck, insufficient blood supply to the placenta and fetal asphyxia), and environment factors (such as high altitude) [1, 4, 5]. PH is also associated with intrauterine growth restriction, fetal brain injury, and premature delivery [6–8]. In addition, PH has long-term adverse health effects on offspring’s health, namely, the nervous system, cardiovascular and metabolic disease, and so on [9–11]. PH can result in cognitive dysfunction and metabolic disease, altered cardiac function, and increased susceptibility to ischemia–reperfusion injury [12–14]. These results are consistent with the fetal origins of adult disease (FOAD) theory proposed by David Barker [15]. The “Barker hypothesis” highlights the importance of intrauterine factors as contributors to FOAD, and recent investigations indicate that adverse intrauterine environments can cause changes in epigenetic modification and result in adult disease [16–18].

Ovarian reserve refers to the total number of primordial follicles remaining in two ovaries, which is established in the fetal period and vulnerable to adverse intrauterine environments. It has previously been observed that ovarian follicle and oocyte development is sensitive to oxygen tension [19]. Disturbance of the intrauterine environment leads to hypoxia and then contributes to an increased risk of ovarian dysfunction [20–23], such as diminished ovarian reserve (DOR). DOR is defined as a decreased number or quality of oocytes in the ovary, accompanied by decreased anti-Mullerian hormone (*AMH*) and antral follicles (*AFC*), and by increased follicle-stimulating hormone (*FSH*). DOR has multiple etiologies including autoimmune, genetic, iatrogenic, and idiopathic etiologies [24]. In addition, many genes are associated with DOR, including growth differentiation factor 9 (*GDF9*) and newborn ovary homeobox gene (*NOBOX*) [25–27]. Based on previous research, Gdf9 was regulated by Nobox [28]. *GDF9* is an oocyte-secreted factor that plays an important role in follicular development [25]. *GDF9* can inhibit the apoptosis and atresia of cumulus cells and regulate the growth and differentiation of early follicles [29, 30]. In vitro experiments showed that Gdf9 knockdown could inhibit the formation of primordial follicles [30]. In addition, recent evidence suggests that mutations and polymorphisms in *GDF9* are associated with DOR [25, 31]. *NOBOX* is an important transcription factor for follicular development [32]. It can directly regulate follicle development genes through its homeodomain combined with specific DNA sequences in early folliculogenesis [33]. In addition, *NOBOX* is an oocyte-specific gene and is expressed in primordial and germ cell cysts [34–36]. Nobox knockout mice showed postnatal oocyte loss [32]. In addition, disruption of the Nobox gene had an effect on folliculogenesis and oogenesis, resulting in ovarian dysfunction [31, 37]. Whether ovarian dysfunction is related to epigenetic modification of Nobox is worth investigating.

The occurrence of FOAD is related to increased susceptibility to certain adult diseases and is closely related to epigenetic modification. It is known that the adverse intrauterine environment can lead to epigenetic changes that would be sustained throughout one’s lifetime, and potentially be passed on to offspring. Epigenetic regulation includes DNA methylation/demethylation, noncoding regulatory RNAs, and histone modifications [18]. Epigenetic modifications regulate gene expression without altering DNA sequences. Epigenetic modifications are responsive to environmental stimuli and could pass on to the next generation [38]. Therefore, alteration of epigenetics triggered by prenatal hypoxia could contribute to an increased risk of FOAD. 5-Hydroxymethylcytosine (5hmC) is the intermediate in the oxidative DNA demethylation pathway. The global 5hmC level is a critical epigenetic mark for ovarian development and aging [39, 40]. Three Tet (ten-eleven translocation) enzymes, namely Tet1–3, can promote DNA demethylation by oxidizing the methyl group of 5-methylcytosine to 5hmC and regulating 5hmC modification dynamics in different genomic loci and cellular contexts [41]. Vitamin C is not only an antioxidant but also a regulator of epigenetic modifications that can enhance the activity of Tet family dioxygenases. Since the addition of vitamin C to mouse ES cells promotes Tet activity, leading to a rapid and global increase in 5hmC, in vitro cell culture models are usually used to study the relationship between 5hmC modification and the target gene [42]. Under normal conditions, 5-methylcytosine (5mC) can be oxidized to 5hmC by the Tets enzyme; however, Tets displayed significant decreases in ovaries exposed to PH, and the association between the alteration of Tets expression and the function of the ovary deserves further study.

At present, there is litter research on the effect of prenatal hypoxia on the reproductive function of offspring, and the specific mechanism remains unclear. Our aims are to study the effect of hypoxia during pregnancy on the Nobox gene of the ovary of offspring and to explore the relationship between the Nobox gene and hydroxymethylation.

This work investigates the epigenetic mechanisms of prenatal hypoxia on Nobox gene functions in ovary development in female offspring. The data may help to further understand the mechanism of the prenatal hypoxia-induced increase in risks in the development of ovarian dysfunction later in life [43].

## Materials and Methods

### Animals

All procedures were approved by the ethical committee of Soochow University. One female rat was mated with one male rat. When vaginal plugs were observed, the day was recorded as embryonic day 1 (E1). The control group (CON, 21% oxygen) and hypoxia group (HY,10.5% oxygen, from E5 to E21) were enrolled in the experiment. A modest 10.5% O_2_ concentration corresponds to high altitude living (> 2,500 m above sea level) at which more than 140 million people currently reside. The hypoxia chamber was fabricated in which the oxygen concentration can be arbitrarily set. The nitrogen generated from the N2^+^ gas generator was mixed with ambient air at an arbitrary ratio using a N2^+^ air blender to generate hypoxia. In this study, we generated a hypoxia model using 10.5% oxygen which has been set up in our lab and demonstrated to be stable [7]. All animals were housed in standardized laboratory conditions (12–12-h light–dark cycle) with free access to feed and water at room temperature. All female SD rats (*n* = 12 per group) give birth naturally, then we selected one female offspring at 3 months from each litter to perform Western blot and q-PCR. As for 5-hmC sequencing, we chose 1–2 female offspring per litter to get more sample size for the experiment. Body weight after birth and at 3 months of age was measured. Each 3-month-old female offspring rat was anesthetized intraperitoneally with chloral hydrate (4%) and sacrificed. Rats underwent cuts into the abdominal cavity with scissors in a midline incision. One ovary of each rat was stored in a − 80 °C freezer, and the other ovary was fixed in formalin-paraldehyde. Unified incineration treatment of animals occurred after euthanasia.

### Estrous Cycle Determination of Offspring Rats

The estrous cycle was observed at 8 weeks of rats for consecutive 10 days. Vaginal cells were collected via saline lavage and then fixed with absolute ethyl alcohol and stained with hematoxylin and eosin (HE). The stages of the estrous cycle were determined based on vaginal cytology.

### Quantification of Plasma AMH, FSH, LH, and T of Offspring Rats

The rat offspring were anesthetized by intraperitoneal injection of chloral hydrate. After exposing the abdominal cavity, approximately 4–5 ml of arterial blood was drawn from the abdominal aorta with a 5-ml syringe. Blood samples were centrifuged at 3000* g* for 10 min immediately after collection. The upper layer of plasma was drawn for later use. Separated plasma samples were stored at − 80 °C until use. The plasma levels of AMH, FSH, luteinizing hormone(LH), and testosterone (T) were measured by a rat AMH ELISA kit (AMH, SBJ-R0231, SenBeiJia, Nanjing, China), a rat FSH ELISA kit (FSH, SBJ-R0930, SenBeiJia, Nanjing, China), a rat LH ELISA kit (LH, SBJ-R0866, SenBeiJia, Nanjing, China), and a rat T ELISA kit (T, SBJ-R0994, SenBeiJia, Nanjing, China) respectively. Assays were performed according to the manufacturer’s instructions. The absorbance at 450 nm was determined using Multiskan Spectrum.

### Ovary Morphology and Numbers of Follicles of Offspring Rats

Ovaries were stained with HE according to standard protocols. To determine ovarian morphology and follicles, every 12th section was mounted on a glass slide, and the number of follicles was counted. Each section was 8 µm. Only follicles with a visible nucleus were counted.

### Cell Culture

Currently, we do not have a suitable ovarian-related cell line. 3T3 cells are mouse embryonic fibroblasts derived from mouse embryos, and we verified that Nobox and Tet were expressed at high levels in 3T3 cells. Therefore, we chose 3T3 cells to investigate the potential relationship between Nobox and Tet in vitro. 3T3 cells may not be the best cell for the study. Nobox and Tet were expressed in 3T3 cells so that they can be used for investigation. 3T3 cells were cultured at 37 ℃ in Dulbecco’s modified Eagle’s medium (DMEM, high-glucose supplement) containing 10% (v/v) heat-inactivated fetal bovine serum (HyClone, UT, USA) and 100 mg/ml penicillin G and 100 mg/ml streptomycin sulfate. After seeding in 6-cm dishes overnight, cells were treated with 0.5 mM l-ascorbic acid under hypoxic condition for 48 h. The expression of Nobox, Gdf9, and Tets was examined by q-PCR and/or Western blot.

### Western Blot Analysis

Ovary tissues (50 mg) or 3T3 cells were disrupted in 500-μl of RIPA buffer supplemented with PMSF and then held on ice for 30 min. After centrifugation at 13800* g* for 30 min at 4 °C, part of the supernatant was extracted, and the protein concentration was measured according to the instructions of the Detergent Compatible Bradford Protein Assay Kit (P0006C-2, Beyotime). The rest of the supernatant was denatured at 96 ℃ for 10 min and subjected to Western blot analysis. Protein (20 μg) was loaded onto 10% SDS-PAGE gels and transferred to PVDF membranes. NOBOX and β-actin protein abundance were measured using primary antibodies (NOBOX, 1:1000, sc-514178, Santa Cruz; β-Actin, 1:5000, ab008-10, Multisciences, China). Blots were visualized using chemiluminescence detection (Amersham Biosciences, Piscataway, NJ, USA). Imaging signals were digitized and analyzed with a UVP imaging system (Tanon-5200, Shanghai, China). The membrane was incubated with a secondary antibody; HRP-conjugated goat anti-mouse IgG (1:5000; GAM0072, LianBio, China) or goat anti-rabbit IgG (1:5000; GAR0072, LianBio, China) was applied in TBS-T for 1 h at room temperature. The relative density of bands was normalized to actin as a control. The gray value was analyzed by Image J software.

### Quantitative Real-time Polymerase Chain Reaction (qRT-PCR)

Total RNA was extracted from offspring ovaries using RNAiso Plus (TaKaRa). The extracted RNA (1000 ng) was reverse-transcribed with a RevertAid First Strand cDNA Synthesis Kit (Thermo Scientific) according to the manufacturer’s instructions. qRT-PCR was performed with gene-specific primers, cDNA and SYBR Premix Ex Taq (TaKaRa), using a Bio-Rad IQ icycler. Primer sequences are listed in Table S1. Each assay was repeated 3 times. The gene expression was normalized to β-actin using the 2^−△△CT^ method.

### Quantification of DNA Methylation and Hydroxymethylation

Ovaries were used for genomic DNA extraction. Genomic DNA was extracted using phenol: chloroform: isoamyl alcohol (25:24:1, Solarbio). Dot blot analysis was performed to detect hydroxymethylation levels. Ten microliters of 100 ng/μl genomic DNA was denatured with 10 μl of 2 M NaOH for 60 min. Two microliters of genomic DNA was spotted on an NC membrane (Millipore). The membrane was dried at RT for 30 min and incubated for 60 min at 75 °C. The membrane was then blocked with 5% BSA in TBS-T for 1 h and incubated overnight with a rabbit anti-5-hmC polyclonal antibody (1:30,000, 39,769, Active) in TBS-T at 4 °C. The membrane was incubated with a goat anti-rabbit IgG secondary antibody (1:5000; GAR0072, Multisciences, China) in TBS-T for 1 h at RT. The dot blot intensity was quantified by a UVP imaging system (Tanon-5200, Shanghai China). The gray value was analyzed by Image J software.

### Hydroxymethylation Level Detection

Five candidate regions of Nobox were selected and sequenced. The selection was based on a previous experiment. We previously performed genome-wide hydroxymethylation sequencing of the rat forebrain. According to the results, we found that the Nobox gene has abundant hydroxymethylation modification sites, so we selected five enriched peaks to carry out target region hydroxymethylation sequencing. The DNA hydroxymethylation level was evaluated at Shanghai Genesky Biotechnology Company (Shanghai, China), with an NGS-based multiple targeted CpG hydroxymethylation analysis method. Briefly, genomic DNA was quantified by Qubit 3 (Invitrogen). With 0.2% synthetic fully methylated and fully hydroxymethylated control DNA spiked in the genomic DNA was treated using T4 β-glucosyltransferase (β-GT, NEB), and APOBEC3A (NEB) in sequence. Enzyme-treated genomic DNA was used as a template for multiplex PCR amplifications with HotStarTaq polymerase (TaKaRa). The PCR program included 95 °C for 2 min; 11 cycles of 95 °C for 20 s, 62 °C for 40 s with a decreasing temperature step of 0.5 °C per cycle, and 72 °C for 1 min; then 24 cycles of 95 °C for 20 s, 64 °C for 30 s, and 72 °C for 1 min; and finally 72 °C for 2 min. PCR products were diluted and amplified using indexed primers with HotStarTaq polymerase (TaKaRa) to generate a sequencing library. The PCR program was 95 °C for 2 min; 12 cycles of 95 °C for 20 s, 60 °C for 40 s, and 72 °C for 1 min; and 72 °C for 2 min. PCR products were separated by agarose electrophoresis and purified using a TIANgel Midi Purification Kit (TIANGEN). Libraries from different samples were quantified, pooled, and sequenced on an Illumina sequencer according to the manufacturer’s protocols, generating 2 × 150 bp paired-end reads with an average depth of 1000 × at the target region.

### Statistical Analysis

The statistical analysis was performed using GraphPad Prism 8. For the statistical data that needed to be *t* tested, we first tested whether the data between the two groups (CON group and PH group) had homogeneity of variance. For the data with an *F* value greater than 0.05, we performed a parametric *t* test, and for the data with an *F* value less than 0.05, we performed a nonparametric *t* test. Data were determined for the difference between 3 groups (CON group, PH group, and VitC group) using 2-way analysis of variance (ANOVA) and *P* < 0.05 was considered significant. Data were expressed as the mean ± SEM. The sample size was chosen on the basis of similar previous studies, and not on statistical methods to predetermine sample size.

## Results

### Changes in Ovarian Function in PH Offspring

**Fig. 1:**
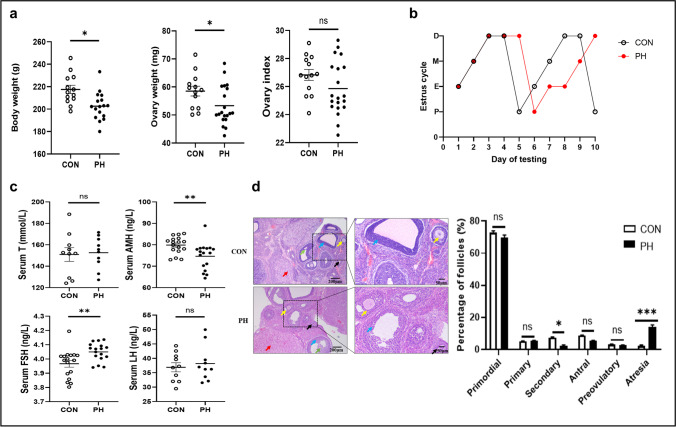
Phenotypes of the rats exposed to PH. **a** Body weight and ovary weight were decreased in the PH group, whereas the ovary index showed no difference between the two groups. (Normoxia group (CON), empty blot, *n* = 10; prenatal hypoxia (PH), black blot, *n* = 10). **b** Representative estrous cycle in two groups. (CON, empty blot, *n* = 10, PH, black blot, *n* = 10). **c** Serum T, AMH, FSH, LH levels. (CON, empty blot, *n* = 10, PH, black blot, *n* = 10). **d** Effect of PH on the ovary morphology and percentage of follicles in rats. (CON, empty column, *n* = 10, PH, black column, *n* = 10). (D, diestrus; M, metestrus; E, estrus; P, proestrus; T, testosterone; AMH, anti-Müllerian hormone; FSH, follicle-stimulating hormone; LH, luteinizing hormone; red arrow, corpus luteum, yellow arrow, primary follicle; green arrow, secondary follicle; black arrow, atretic follicle; blue arrow: granule cells). *P < 0.05, **P < 0.01, ***P < 0.001 PH vs. CON

Litter size was not different between the control and hypoxic groups (*P* > 0.05, *t* test). Female survival ratios of the control group were 98.3% (57/58). Female survival ratios of the hypoxia group were 94.5% (52/55). Female survival ratios were not different between the control and hypoxic groups (*P* > 0.05, chi-square). Birth body weight was measured at birth. Bodyweight and ovary weight were measured at 3 months. Birth body weight was reduced by 20.3% in the PH group (*P* < 0.0001, *t* test). Bodyweight at 3 months was reduced by 3.82% in the PH group (*P* > 0.05, *t* test). Ovary weight at 3 months was reduced by 5.27% in the PH group (*P* > 0.05, *t* test) (Table S2). Bodyweight and ovary weight at 3 months were reduced in the PH group compared with the control group (*P* < 0.05, *t* test); however, there was no significant difference in the ratio of ovary weight to body weight between the two groups (Fig. 1a). In addition, as shown in Fig. 1b, PH rat exhibited disrupted estrous cycles as compared with control rats. Specifically, the estrus period and diestrum period are prolonged in PH group. The serum AMH level was significantly decreased (*P* < 0.01, *t* test), whereas the serum FSH level was significantly increased in the PH group (*P* < 0.01, *t* test). In addition, the LH level and T level were not significantly different between the two groups (Fig. 1c). The number of secondary follicles was decreased in PH rats (*P* < 0.05, *t* test). In addition, the number of atresia follicles was increased in PH rats (Fig. 1d) (*P* < 0.001, *t* test). The numbers of other follicles were similar in PH and control rats. These data indicated that ovarian function in the offspring of the PH group was obviously abnormal.

### Nobox Was Downregulated in the Ovaries Exposed to PH

**Fig. 2:**
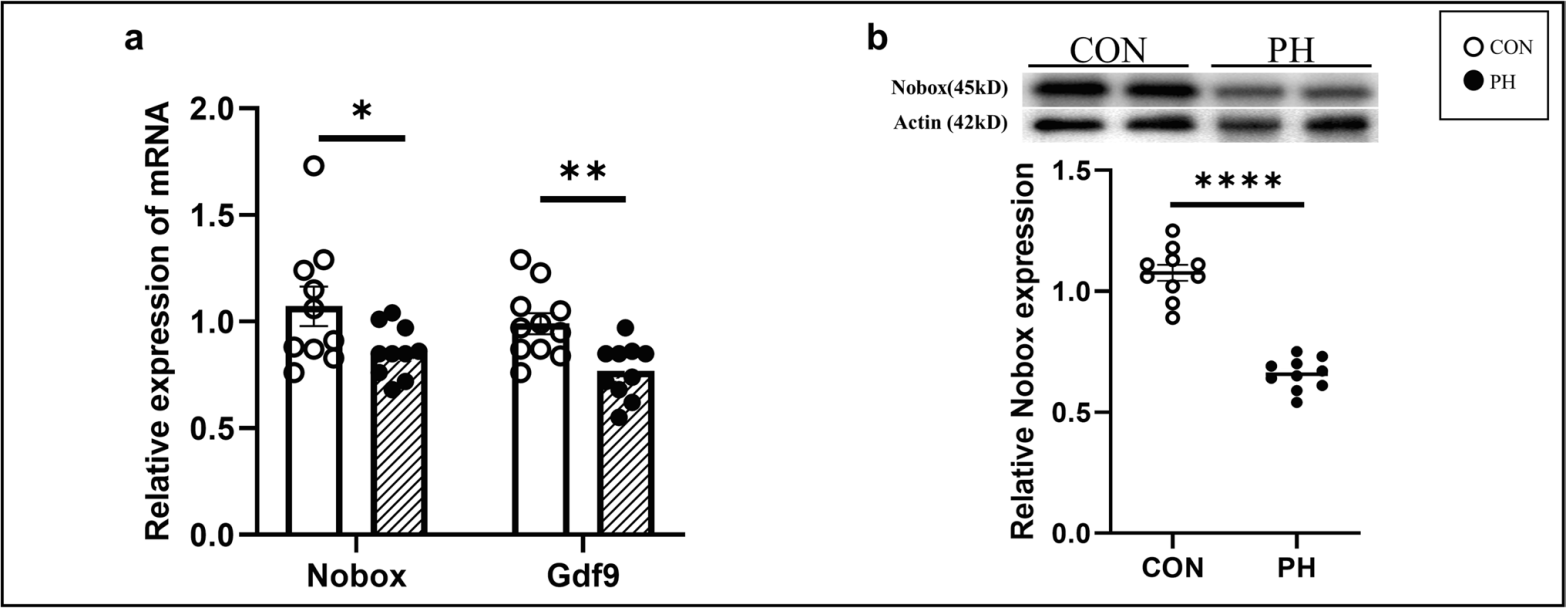
Expression of Nobox and Gdf9 in the ovaries of female offspring. **a** Nobox and Gdf9 mRNA levels in the ovaries. (Control group (CON), empty dot, *n* = 10, prenatal hypoxia (PH), black dot, *n* = 10). **b** NOBOX protein levels in the ovary. (CON, empty dot, *n* = 10, PH, black dot, *n* = 10). *P < 0.05, **P < 0.01, ****P < 0.01

The q-PCR results showed that the mRNA levels of Nobox (*P* < 0.05, *t* test) and Gdf9 (*P* < 0.01, *t* test) in the ovary were decreased in the PH group (Fig. 2a). NOBOX protein was decreased in the ovaries of the PH group (*P* < 0.0001, *t* test) (Fig. 2b). These results showed that Nobox was downregulated in the ovary exposed to PH.

### Prenatal Hypoxia Resulted in Hypo-Hydroxymethylation in the Ovary Exposed to PH

**Fig. 3:**
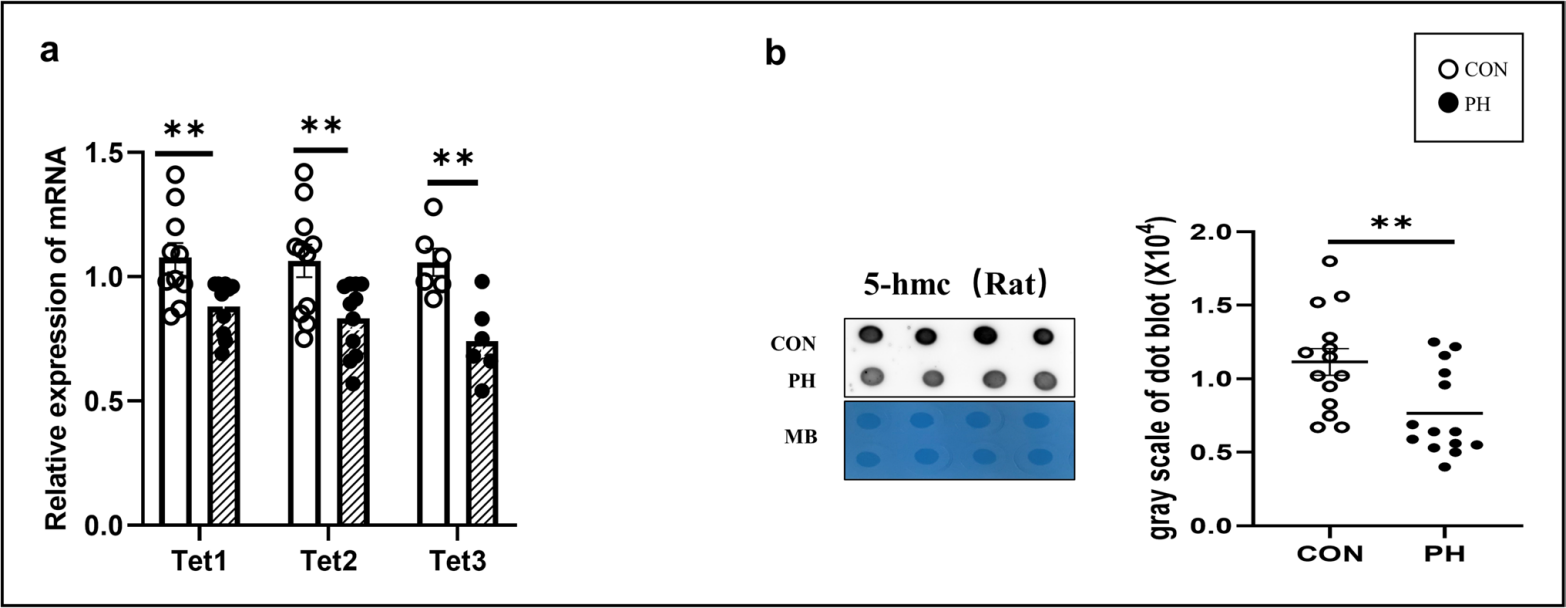
Tets and DNA hydroxymethylation levels in the rat ovary. **a** Tet1, Tet2, and Tet3 mRNA levels in the ovary. (CON, empty dot, *n* = 10, PH, black dot, *n* = 10). **b** Dot blot analysis was used to detect the 5-hmC level in rat ovaries. MB staining was used as a loading control. The grayscale of dot blot results in the two groups was compared. (Normoxia group (CON), empty dot, *n* = 10, prenatal hypoxia (PH), black dot, *n* = 10). **P < 0.01

In addition to changes in ovarian genes, PH can also cause changes in ovarian hydroxymethylation. Therefore, we detected the levels of Tets, which catalyze the successive oxidation of 5mC to 5hmC, in ovaries exposed to PH. The q-PCR results showed that the expression of Tets in the ovary was decreased in the PH group (*P* < 0.01, *t* test) (Fig. 3a). The dot blot results showed that the 5hmC levels were decreased in the PH group (*P* < 0.01, *t* test) (Fig. 3b).

### Decreased Hydroxymethylation Level of the Nobox Gene in the Ovary Exposed to PH

**Fig. 4:**
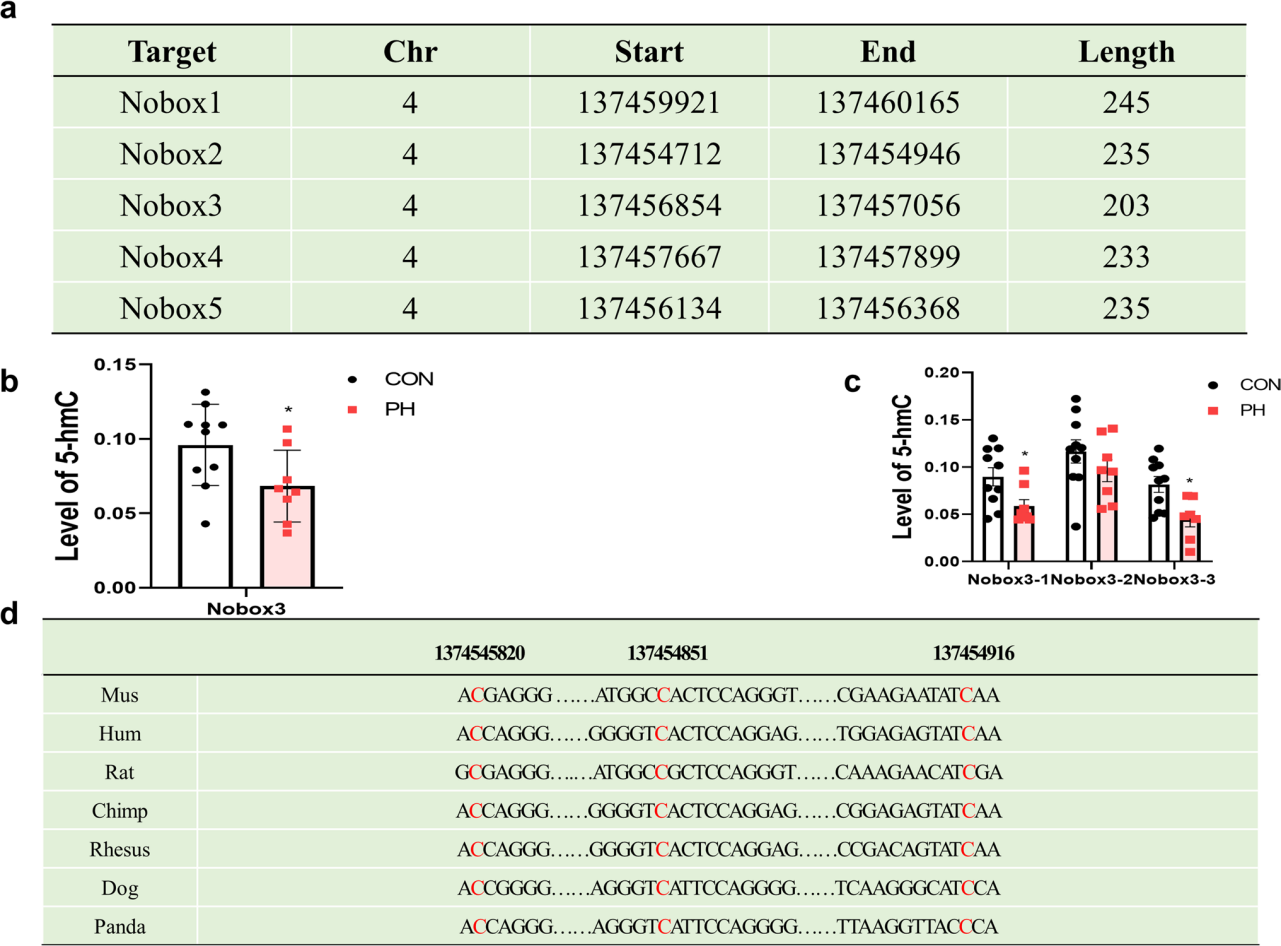
DNA hydroxymethylation level detection of rat ovaries. **a** Five candidate 5hmC modification regions (1–5) of Nobox were selected. **b** Candidate 5hmC modification region 3 of Nobox was decreased in PH. **c** 5hmC levels of three fragments of candidate 5hmC modification region 3 exposed to PH. **d** DNA sequence alignment among different species of the candidate 5hmC modification region of Nobox. (Normoxia group (CON), black dot, *n* = 10, prenatal hypoxia (PH), red dot, *n* = 10). *P < 0.05

To identify whether Nobox gene expression is regulated by hydroxymethylation modification, high-throughput sequencing was performed on the Nobox gene. Five candidate regions of Nobox were selected and sequenced (Fig. 4a). The third region of Nobox showed a significant difference in the ovary exposed to PH. (Fig. 4b, c) (*P* < 0.05, *t* test). These results suggested that Nobox was hypo-hydroxymethylated in the ovary exposed to PH. Figure 4d shows that DNA sequence alignment from various species demonstrated the conservation of those nucleotides.

### Downregulation of Nobox, Gdf9, and Tets in 3T3 Cells Treated with Hypoxia

**Fig. 5:**
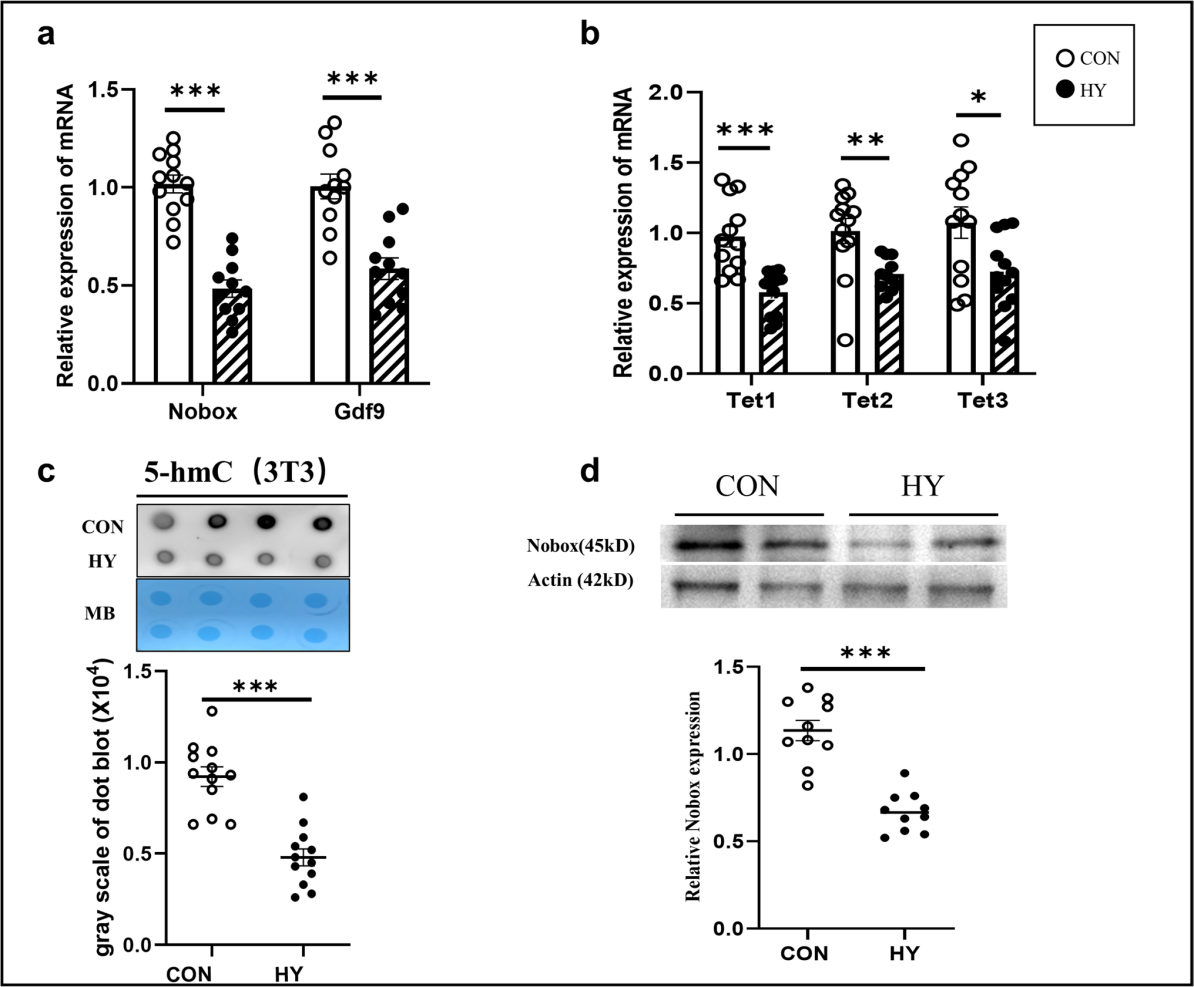
Expression of Nobox, Gdf9, Tets, and global 5hmC levels in 3T3 cells treated with hypoxia. **a** Nobox and Gdf9 mRNA levels in 3T3 cells treated with hypoxia. (Normoxia group (CON), empty dot, *n* = 10, hypoxia (HY), black dot, *n* = 10). **b** Tet1, Tet2, and Tet3 mRNA levels in 3T3 cells exposed to PH. (CON, empty dot, *n* = 10, HY, black dot, *n* = 10). **c** Dot blot analysis was used to detect the 5-hmC level in 3T3 cells treated with hypoxia. MB staining was used as a loading control. The grayscales of dot blot results were compared. (CON, empty dot, *n* = 10, HY, black dot, *n* = 10). (d) NOBOX protein levels in 3T3 cells treated with hypoxia. CON, empty dot, *n* = 10, HY, black dot, *n* = 10. *P < 0.05, **P < 0.01, ***P < 0.01

To further explore the possible association between NOBOX and TETs, 3T3 cells were treated with hypoxia. 3T3 cells were cultured with Vitc (A5960, Sigma) in a concentration range from 0.05 mM to 1 mM under hypoxic conditions for 48 h. Tets mRNA expression was evaluated by q-PCR to determine the best working concentration of Vitc. The rational dosage of 3T3 cells was 0.5 mM under hypoxic conditions for 48 h as the expression of Tet1 increased significantly [44]. The q-PCR results showed that the mRNA expression of Nobox and Gdf9 was decreased in 3T3 cells treated with hypoxia (*P* < 0.001, *t* test) (Fig. 5a). Tet1 (*P* < 0.001, *t* test), Tet2 (*P* < 0.01, *t* test), and Tet3 (*P* < 0.05, *t* test) were decreased in 3T3 cells treated with hypoxia (Fig. 5b). The dot blot results showed that the global 5hmC levels were decreased in the hypoxia group (*P* < 0.001, *t* test) (Fig. 5c). The protein level of NOBOX was downregulated in 3T3 cells treated with hypoxia (*P* < 0.001, *t* test) (Fig. 5d).

### Upregulation of Nobox, Gdf9, and Tets in 3T3 Cells Treated with Vitamin C

**Fig. 6:**
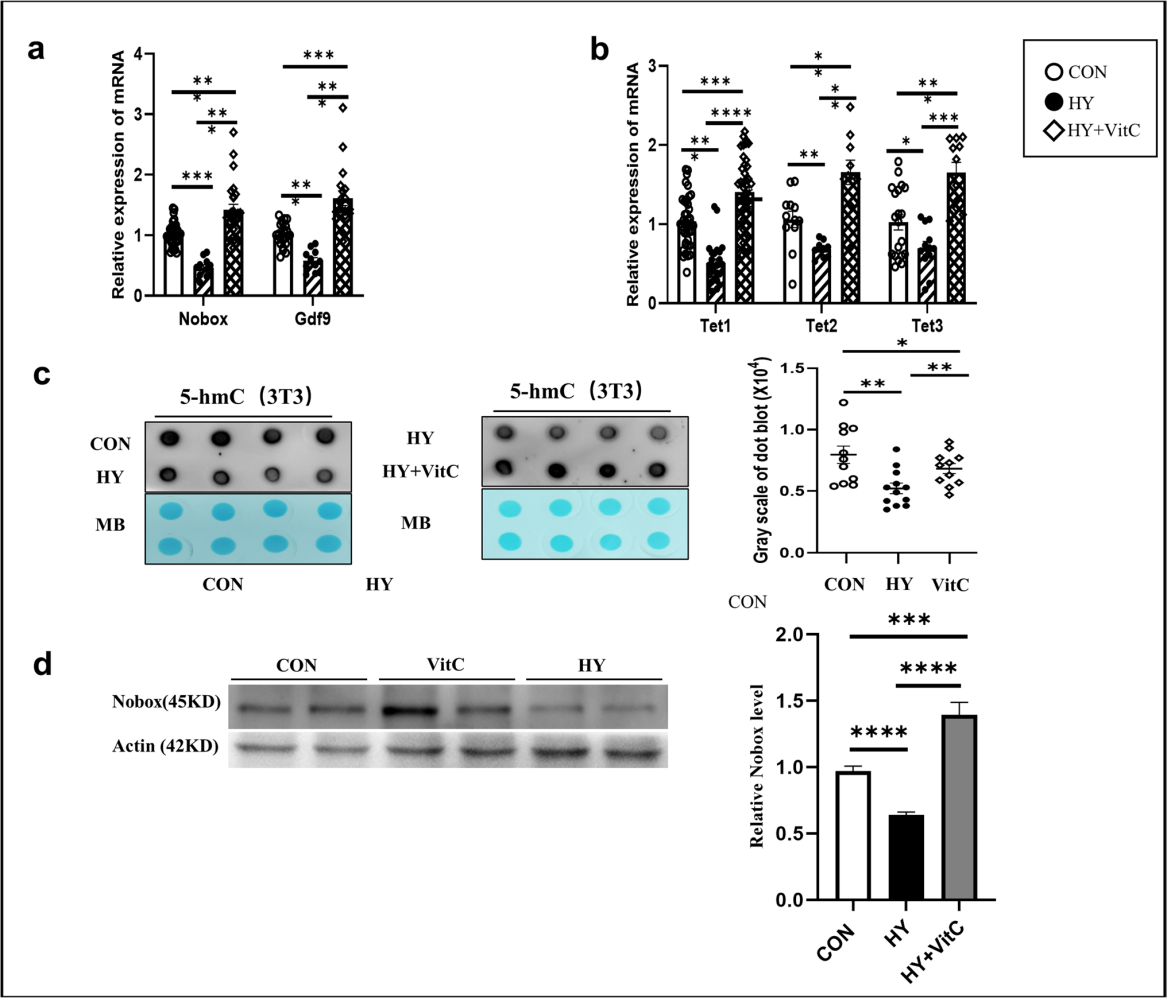
Expression of Nobox, Gdf9, Tets, and global 5hmC levels restored in 3T3 cells treated vitamin C. **a** Nobox and Gdf9 mRNA levels in 3T3 cells treated with vitamin C. **b** Tet1, Tet2, and Tet3 mRNA levels in 3T3 cells treated with vitamin C. **c** Dot blot analysis was used to detect the 5-hmC level in 3T3 cells treated with hypoxia. **d** NOBOX protein levels in 3T3 cells treated with hypoxia and vitamin C. (Normoxia group (CON), empty dot, *n* = 10, hypoxia (HY), black dot, *n* = 10, VitC group (VitC), empty rhombus, *n* = 10). *P < 0.05, **P < 0.01, ***P < 0.01, ****P < 0.01

To further explore the possible mechanisms of Nobox and Tets, 3T3 cells were treated with vitamin C, a coactivator of Tets. q-PCR showed that the mRNA expression of Gdf9 (*P* < 0.01, ANOVA) and Tets (Tet1 *P* < 0.0001, Tet2 *P* < 0.05, Tet3 *P* < 0.001, ANOVA) was increased in 3T3 cells treated with vitamin C (Fig. 6a, b). The dot blot results showed that global 5hmC was decreased in 3T3 cells treated with hypoxia, but increased after treatment with vitamin C (*P* < 0.01) (Fig. 6c, t test). The protein level of NOBOX was downregulated in 3T3 cells treated with hypoxia, but restored after treatment with vitamin C (*P* < 0.001, *t* test) (Fig. 6d).

## Discussion

In the present study, we used a rat model to show the effects of PH on the ovary. Birth weight was reduced by 1.28 g and 20.3% by PH [7], which indicated that PH affected not only general growth but also ovarian function [45]. These alterations are mediated via programmed changes in the hypothalamic-pituitary–gonadal axis and the structure and function of the ovaries. These results were consistent with a previous study showing that chronic gestational hypoxia accelerated ovarian aging and lowered ovarian reserve [43]. The current study found that treatment with PH induced ovarian dysfunction, including disrupted estrous cycles, hormonal abnormalities, decreased secondary follicles, and increased atresia follicles. In addition, we observed that Nobox, Gdf9, Tet1, Tet2, and Tet3 were significantly decreased in the ovaries of the PH group. By performing hydroxymethylation level detection, decreased hydroxymethylation levels of Nobox were found in the PH group. Furthermore, we chose 3T3 cells to investigate the potential relationship between Nobox and Tet in vitro, because we discovered that Nobox and Tet were expressed at high levels in 3T3 cells and that we did not have a suitable ovarian cell line. In 3T3 cells treated with hypoxia, the mRNA expression of Tet1, Tet2, and Tet3 was decreased, while that of Nobox and Gdf9 was decreased. After treatment with vitamin C, the mRNA expression of Tet1, Tet2, Tet3, Nobox, and Gdf9 was increased. The changes in the 3T3 cells indicated that hydroxymethylation could be reversed after treatment with vitamin C [42] and possible association between Nobox and Tets in the two groups.

Ovarian function includes reproductive function and endocrine function, which begin to form in the fetal period. A favorable in utero environment is a necessary condition for embryo development and ovarian development. Oxygen tension is important for ovarian follicular reserve at 3 months of gestation, because primordial germ cells migrate to the genital ridge and follicular development depends on oxygen tension [46]. Exposure to PH may have significant impacts on ovarian function including ovarian reserve, estrous cycle, fertility, and sex steroid hormone levels. As reported before, ovarian dysfunction was likely to be associated with an early decline in follicles exposed to PH [42]. In our study, disrupted estrous cycles, hormonal abnormalities, and disordered follicular development could be caused by PH.

*NOBOX* is essential for the early development of the ovary and is involved in the regulation of genes at the transcription level during the follicle stages [47]. Mutation of *NOBOX* was reported in women with POI and infertility [37]. Nobox knockout female mice are infertile and loss of Nobox leads to the downregulation of Gdf9 and the expression of other oocyte-specific genes [32, 36]. The Gdf9 gene regulates granulosa cell mitosis to influence the molecular dialog between the surrounding somatic cells and the oocyte [48, 49]. In a previous study, altered expression of Nobox and Gdf9 induced ovarian dysfunction [42, 50–53]. In our study, the expression of both Nobox and Gdf9 was decreased in the PH rat ovary and associated with ovarian dysfunction.

Ten-eleven-translation 5-methylcytosine dioxygenase (Tets) is involved in epigenetic regulation of hydroxymethylation. The Tet family of enzymes catalyzes the conversion of 5mC to 5hmC, a modified cytosine base that facilitates gene expression. It has been demonstrated that Tet is essential for viability and fertility. Meanwhile, the enzymatic activities of Tets require oxygen. In our study, Nobox changed dynamically with Tets in ovaries exposed to PH. To identify whether Nobox gene expression is regulated by hydroxymethylation modification, high-throughput sequencing was performed with the Nobox gene. Because the Nobox gene does not have a CPG island, methylation sequencing of the Nobox gene was not performed. The sequencing results suggested that Nobox was hypo-hydroxymethylated in the ovary exposed to PH. In addition, an in vitro experiment using 3T3 cells treated with hypoxia indicated significant decreases in Tets and Nobox. The dominant form of vitamin C is ascorbate. It serves as a direct cofactor for Tets that catalyzes the oxidation of 5mC into 5hmC. Treatment with vitamin C enhanced Tet expression and increased the expression of Nobox and the known downstream gene Gdf9. Therefore, vitamin C was useful to reverse the decreased 5hmC following exposure to adverse in utero environments and prevent adverse outcomes caused by abnormal pregnancy. This strongly suggested that the Nobox gene could be regulated by 5hmC modification. Prenatal vitamin C supplementation may have a long-term effect on FOAD in general. In the future, more downstream genes of Nobox will be explored to clarify the effect of hypoxia on ovarian function.

**Fig. 7:**
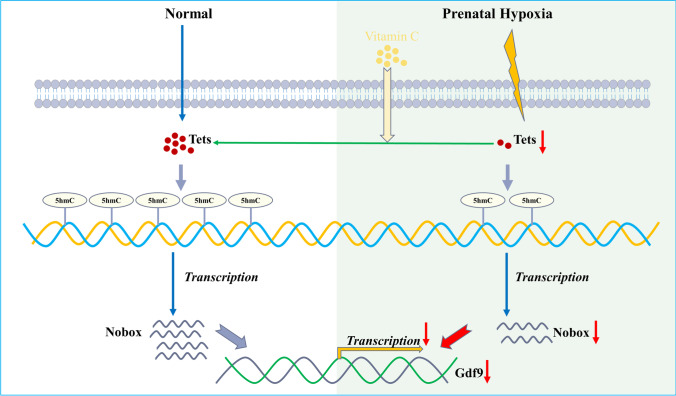
Hypo-hydroxymethylation of Nobox is associated with ovarian dysfunction in rat offspring exposed to prenatal hypoxia. Adverse intrauterine environments, such as hypoxia, are closely linked to ovarian dysfunction. Tets decreased in the offspring female rats exposed to hypoxia, accompanied by a decrease in hydroxymethylation (5hmC) modification. As downregulated Tets was associated with downregulated Nobox and Gdf9, Tets is considered an epigenetic gene involved in ovarian dysfunction. The expression of Nobox, Gdf9, and Tets was decreased in 3T3 cells treated with hypoxia, but restored after treatment with vitamin C. Vitamin C could be a potential treatment to prevent ovarian dysfunction. This study will provide new insight for the identification of novel biological markers and treatment of ovarian dysfunction

In summary, PH could affect gene expression through epigenetic regulation in a long turn, and abnormal 5hmC levels of Nobox caused by PH were a major cause of ovarian dysfunction in rat offspring exposed to PH (Fig. 7). Vitamin C could be a potential treatment to prevent ovarian dysfunction following exposure to PH.

### Research and Clinical Implications

The present findings support the hypothesis that Nobox and Tets play a vital role in the onset of ovarian dysfunction. Further work on the involvement of Nobox and Tets in ovarian dysfunction pathogenesis may be promising to inspire novel therapies, particularly in patients with ovarian dysfunction and infertility.

### Limitations

We did not check the pregnant rats for body weight, water, food intake, cross-fostering, litter size, or glucocorticoid levels, which were fundamental flaws in the study design and might induce differences in energy intake during the lactation period. In addition, no ovarian cells were used in the study, which may limit the generalizability of these results. Finally, further experiments are clearly warranted to study the exact mechanism by which Nobox acts.

## Conclusion

In conclusion, prenatal hypoxia significantly affected ovary function in offspring and caused downregulation of Tets, which might decrease Nobox/Gdf9 through DNA hydroxymethylation. The evidence showed that epigenetic regulation of Nobox could be one of the possible mechanisms contributing to the dysfunction of ovaries exposed to prenatal hypoxia. The results from prenatal hypoxia experiments indicated impaired hydroxymethylation levels of the genomic DNA in the ovary. This view contributed to further understanding the mechanisms underlying diminished ovarian reserve and suggested the benefits of further investigation of the early prevention of ovarian dysfunction.

## Supplementary Information

Below is the link to the electronic supplementary material.

Supplementary file1 (PDF 91 KB)

Supplementary file2 (PDF 84 KB)
