# Supplementary material for: Hypo-Hydroxymethylation of Nobox is Associated with Ovarian Dysfunction in Rat Offspring Exposed to Prenatal Hypoxia
Source: Reprod Sci. 2022 Mar 7;29(5):1424–36. doi: 10.1007/s43032-022-00866-6 (PMC9005429; doi:10.1007/s43032-022-00866-6)
Supplement: Supplementary file 1 — Supplementary file1 (PDF 91 KB) [file 43032_2022_866_MOESM1_ESM.pdf]

S1 Primer of Nobox, Gdf9, Tet1, Tet2 and Tet3 in rats and mice

Primer	Sequence (5'-3')
Rat Nobox_F	TGCTTGCTTGGTAATCCTGAGG
Rat Nobox_R	AAGCTCCTCAAGTGCTACCTCC
Rat Gdf9_F	TGCTACCAAAGAGGGGGTTCC
Rat Gdf9_R	TGGAAGACATGGGCTCCTTTAC
Rat Tet1_F	CACCTCACGGGTACAGGTTACA
Rat Tet1_R	GGGCCATTTACTGGTTTGTTGT
Rat Tet2_F	CACTTGCAAAACCTGGCTACTG
Rat Tet2_R	CCCAAACGGCATTCTATAGCTC
Rat Tet3_F	CTACGAAGAGTCTGCTGGACACA
Rat Tet3_R	GGCCTTCATCCTTCTCCACTATT
Rat ACTIN_F	CCTAAGGCCAACCGTGAAAAG
Rat ACTIN_R	GCTCGAAGTCTAGGGCAACATAG
Mouse Nobox_F	AACTGGCTGCTTGCTTGGTAGT
Mouse Nobox_R	TTCAAACTCCTCAAGTACTGCCTC
Mouse Gdf9_F	ACCTCTACAATACCGTCCGGC
Mouse Gdf9_R	AAGAGGCAGAGTTGTTCAGAGTG
Mouse Tet1_F	CCAGGAAGAGGCGACTACGTT
Mouse Tet1_R	TTAGTGTTGTGTGAACCTGATTTATTGT
Mouse Tet2_F	ACTTCTCTGCTCATTCCCACAGA
Mouse Tet2_R	TTAGCTCCGACTTCTCGATTGTC
Mouse Tet3_F	GAGGCTTTGCTGGGACAATC
Mouse Tet3_R	CATGGCCTTCCGTGTTCCTA
Mouse GAPDH_F	GGCTGTATTCCCCTCCATCG
Mouse GAPDH_R	CCAGTTGGTAACAATGCCATGT
