# Supplementary material for: Hypo-Hydroxymethylation of Nobox is Associated with Ovarian Dysfunction in Rat Offspring Exposed to Prenatal Hypoxia
Source: Reprod Sci. 2022 Mar 7;29(5):1424–36. doi: 10.1007/s43032-022-00866-6 (PMC9005429; doi:10.1007/s43032-022-00866-6)
Supplement: Supplementary file 2 — Supplementary file2 (PDF 84 KB) [file 43032_2022_866_MOESM2_ESM.pdf]

S2 The weight of female offspring in CON and PH group

	CON ($\bar{X}$±S)	PH ($\bar{X}$±S)
Birth body weight	6.29±0.04	5.01±0.05
Body weight at 3 months	217.2±2.98	208.9±3.03
Ovary weight at 3 months	58.04±1.37	54.98±1.53
